# Recyclability Definition of Recycled Nanofiltration Membranes through a Life Cycle Perspective and Carbon Footprint Indicator

**DOI:** 10.3390/membranes12090854

**Published:** 2022-08-31

**Authors:** Jorge Senán-Salinas, Junkal Landaburu-Aguirre, Raquel García-Pacheco, Eloy García-Calvo

**Affiliations:** 1 IMDEA Water Institute, Avenida Punto Com, 2, Alcalá de Henares, 28805 Madrid, Spain; 2 Chemical Engineering Department, University of Alcalá (UAH), Ctra. Madrid-Barcelona Km 33.600, 28871 Alcalá de Henares, Spain; 3 BETA Technological Center, University of Vic—Central University of Catalonia, Ctra. De Roda, 70, 08500 Vic, Spain; 4 Laboratory of Chemical and Environmental Engineering (LEQUIA), Institute of the Environment, University of Girona, 17003 Girona, Spain

**Keywords:** circular economy, life cycle assessment, carbon footprint, recycling, reverse osmosis membranes, specific energy consumption, modelling, recycled nanofiltration membranes

## Abstract

The direct end-of-life recycling of reverse osmosis membranes (RO) into recycled nanofiltration (r-NF) membranes has been pointed out as a circular technology. For the first time, an environmental analysis of the whole life cycle of r-NF membranes was performed, focused on their usage. The carbon footprint (CF) of NF water treatment processes (Functional Unit: 1 m^3^ of treated water) with different pressure vessel (PV) designs and energy sources using r-NF and commercial NF-270-400 was quantified. Moreover, to compensate for the lower permeability of the r-NF, two design strategies were assessed: A) an increment in inlet pressure, and B) an increase in the number of modules. The inventory included energy modelling for each design and membrane. The interaction of both strategies with the permeability and service life of r-NF, together with different energy sources, was assessed using a novel hybrid analytical–numerical method. The relevance of energy use at the usage stage was highlighted. Therefore, r-NF permeability is the foremost relevant parameter for the definition of CF. The low impact of the r-NF replacement favoured strategy B. The use of an environmental indicator (CF) made it possible to identify the frontiers of the recyclability and applicability of r-NF membranes.

## 1. Introduction

The Circular Economy (CE) has emerged, revitalizing old concepts such as sustainability. This concept aims to transition from current linear model economies into circular ones as a solution to the environmental multicrisis [1] via the minimisation of resource extraction and pollution generation, enhancing the internal recycling of energy and material flows within economies and societies. Closed-loop recycling is the foremost known strategy of this alternative model [2]. However, other strategies, such as open-loop recycling or redesign of the product, are also included to achieve the abovementioned goal. Although these strategies are considered CE strategies to reduce the global environmental impact across societies, a specific analysis must be performed to prevent hidden and undesired side-effects that may result. For that purpose, Life Cycle Assessment (LCA) methodology was pointed out as the most suitable methodology [3]. It is worth remarking on the mention of LCA in the Directive on Waste (EC/2008), where the Life Cycle perspective is introduced as a criterion for the definition of recyclability [4].

The application of LCA in the assessment of emerging recycling technologies was one of the first uses of this methodology [5]. Since the early use of the methodology, recycling technologies across several economic sectors have been assessed [5]. There are two main aspects to consider in the use of LCA over emerging recycling technologies: (i) the introduction of particularities of these technologies or products, and (ii) the management of uncertainty related to low technology readiness levels [6,7]. The perspective of reusing secondary products (the recycling output) is also specific to energy-related sectors. Undoubtedly, a deep understanding of LCA technologies by practitioners influences the perspective of the assessment and, at the same time, reflects the versatility of the methodology. 

In the present article, the main object of the analysis is the direct recycling of end-of-life (EoL) reverse osmosis (RO) membrane modules into nanofiltration (NF) membranes. RO and NF are elements of membrane technologies used for water treatment. Desalination, water reuse and industrial processes are the most common applications [8]. RO membranes allow the separation of ions and particles from the water and the production of freshwater. However, the flow generated by these membranes is significantly reduced over time due to fouling issues. It is estimated that RO membranes are discarded after 5–10 years in landfills, creating thousands of tons of waste annually [9]. Consequently, several recycling alternatives were developed in the last years [10,11,12]. Nonetheless, the most advanced technologies (highest technology readiness level—TRL) are direct RO membrane regeneration (keeping RO performance) and membrane recycling into NF and ultrafiltration (UF) membranes for water treatment [13]. So far, to the knowledge of the authors, these initiatives are being implemented at a pilot scale (NF, UF) [14]. Membrane direct recycling into NF consists of a chemical attack with sodium hypochlorite that partially attacks the PA layer of the RO membrane [15]. As expected, the selectivity reduces significantly for monovalent ions such as Na^+^ or Cl^−^, while the rejection of divalent ions remains significant, achieving rejection coefficients comparable to commercial NF models [14,15]. Unlike RO membranes, the range of performance of NF membranes is wide: between 50% and >90% for divalent ion rejection and between 10% and 95% for monovalent ion rejection [16]. 

A few studies analysed the environ-economic potential of direct recycling into NF and UF [17,18,19]. These works introduced LCA to identify hotspots in the supply chain and processes as well as to account for the environmental balance of recycling. Senán-Salinas et al. conducted an attributional LCA integrating the environmental credits of the substitution and referring to the ILCD guidelines [20]. Through this work, a substitutability factor (SF) was developed to correct the differences in performance between recycled modules and their commercial counterparts. The SF included permeability and lifespan. However, due to the lack of information regarding quantification of the lifespan, it was necessary to develop new indicators based on the Life Cycle Impact Assessment results quantified in this way: the thresholds of the lifespan at which the recycled membranes would achieve overall environmental balance. The design of these new indicators and analysis allowed for a first inference of activities where the use of recycled membranes could mean a reduction in the environmental impact of membrane replacement in the activity. With the lower impact of recycled membranes compared to that of the production of new membranes, activities with a high replacement rate (for instance, landfill leachate treatment) were identified as the preferable ones [17,18]. Nonetheless, there are still some aspects of the recycling processes and products that have not hitherto been addressed.

Previous works identified energy use as the major contributor to the environmental impact of membrane processes [21,22,23,24]. Specific Energy Consumption (SEC) indicates how much energy is needed to produce 1 m^3^ of product water. The SEC of brackish water (BW) RO desalination is approximately 0.4–3 kwh·m^−3^ and that of seawater (SW) RO desalination is approximately 2.5–4 kwh·m^−3^ [25,26,27]. The value depends on the water quality, membrane properties, and system design, among others [27,28]. However, permeability is one of the most important factors in the definition of the SEC in membrane processes [27]. SEC is also importantly affected by the design. The use of pressure vessels (PV) in RO and NF reduced the SEC of the processing, which constrained the spread of these technologies due to their high energy use [29,30]. Another alternative to reduce SEC impact is to reduce the unitary impact of the energy through the hybridisation of membrane technologies with renewable energies [31]. Nonetheless, this has several technical and economic barriers to resolve [25]. Furthermore, some authors refer to the decoupling of pressure-driven technologies from energy use. The use of gravity-driven (GD) systems was also researched and identified as a suitable alternative, though constrained [24,25].

Although previous papers have quantified the environmental impact of r-NF on recycling processes, to the knowledge of the authors, there are no papers focused on the use of r-NF. The pieces of evidence indicating lower permeabilities in r-NF compared to their commercial counterparts suggest the study of the influence of this parameter on the overall impact of r-NF membranes. These analyses are enhanced by the abovementioned Directive on Waste (EC/2008). This directive promotes the use of LCA at early stages to assess recycled products and identify potential hidden impacts along their whole life-cycle, including at the use stage [4]. The main reasons for these analyses are the prevention of impacts and environmental protection. Therefore, for r-NF, environmental analysis with a whole life-cycle perspective, with special attention to energy use during the water treatment processes, remains a knowledge gap. 

The present article aims at identifying the recyclability and technological niches for converting EoL-RO modules into r-NF through one LCA indicator (GWP or CF) and a holistic life-cycle perspective. Particularly, this study assesses (i) which permeabilities and lifespans limit the implementation of r-NF membranes, and (ii) under which operational conditions (i.e., feed pressure) and designs (i.e., the number of consecutive modules in a stage) the r-NF membranes have an advantageous outcome in environmental terms. Therefore, the novelty of this work is the usage of energy modelling to understand the interaction of membrane performance with system design, to propose an optimal configuration for r-NF. Finally, the article explores a methodological framework where the life cycle perspective and LCA indicators can be used to define recyclability according to the Directive on Waste and CE principles [3,4].

## 2. Materials and Methods

### 2.1. Overal Methodology 

In the present article, firstly, the quantification of CF across the whole life cycle of r-NF was illustrated and compared with a commercial counterpart, NF-270-400 (simplified as NF-270 in the present article), under specific operational conditions. A specific recycled and characterised module at the pilot scale was used for case study analysis (Section 2.4). Secondly, for exploratory analysis, a novel hybrid analytical-numerical analysis was performed for combining energy modelling and LCA-based indicators under a wide range of operational conditions (Section 2.5). The analysis focused on identifying the limits of the membrane properties (L and service life (SL)), system design (number of modules (n), inlet flow (Q_in_) and water recovery rates (WR)), energy source, and design strategies (hereinafter explained). 

This article also assesses two different design strategies to reach the water production of a commercial NF membrane, as r-NF membranes have lower permeability (L) than commercial ones [14]:

- **Strategy A:** Pressure increments with an equal number of elements. This strategy maintains the number of modules and compensates for the lower permeability with increments of Pr_in_. Therefore, the expected SEC of r-NF membranes is higher. This strategy would represent the substitution of commercial elements with r-NF modules in existing systems.
- **Strategy B:** Incrementing the number of elements. Within this strategy, low permeability is compensated for via increments of the active areas within the PV, that is, an increment of the number of elements that are consecutively installed in each stage. This increment of elements (and thus membrane active area) compensates totally or partially for Pr_in_. This strategy cannot be easily adapted to existing systems where PVs are constrained to a particular module capacity. However, it is relevant to explore whether new designs for r-NF systems could minimise and mitigate their energy impact.

These strategies were compared in the case study analysis as well as in the hybrid analytical–numeric analyses. 

### 2.2. Life Cycle Assessment

The present paper refers to CF as the indicator and result of the application of the global warming potential (GWP) or climate change category (as described in Section 2.2.4). Therefore, terminology and structure follow LCA standards ISO 14000:2006 and ISO 14040:2006 [26,27]. 

#### 2.2.1. Goal and Scope 

The main goal of LCA is the comparative analysis of the environmental impact of the membranes considering the whole life cycle. Therefore, the analysis is oriented to the water treatment process, with the functional unit being 1 m^3^ of treated water. This assessment allows for the comparison of different designs (Q_in_, WR and n) as well as their interaction with different membranes (commercial NF-270 vs. r-NF). 

#### 2.2.2. System Boundaries 

**Figure 1:**
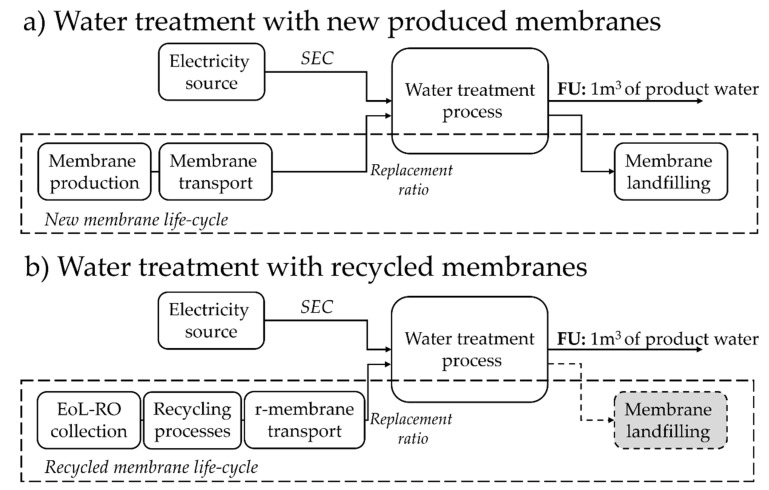
System boundaries and main foreground processes: (**a**) NF process with commercial NF membranes, and (**b**) NF process with r-NF membranes Grey boxes contain compensated impacts due to recycling.

Figure 1 describes the system boundaries of the analyses. The assessment was oriented to a theoretical treatment process where two main factors were considered: the overall impact of the process (Imp_total_) is the sum of the impacts of the replacement (Imp_rep_, developed in Equation (S.1)) and the energy use (Imp_e_, developed in Equation(S.2)), as described in Equation (1), for both membranes: NF-270 and r-NF. Other impacts, such as the infrastructure or cleaning agents, were excluded.

(1) $${\mathrm{Imp}}_{\mathrm{total}}{=\mathrm{Imp}}_{\mathrm{rep}}{+\mathrm{Imp}}_{e}$$

In addition, the impact of commercial membrane replacement was estimated in Equation (S.1) (Supplementary File). This includes the production of the membrane, landfilling, and transport processes. In the case of r-NF, the burdens come from the collection of the EoL-RO modules, their characterisation, the recycling process, and distribution (as in Equation (S.2)). The landfilling of r-NF at its EoL was considered compensated for by the credits of avoiding EoL-RO membrane disposal because of the recycling activity. The allocation of credits to the recycled product is a practice from previous works and is also recommended for the substitution approach under ILCD guidelines [17,32] The number of hours assumed to be worked per year of SL was 6,000 h. This represents 69% of the length of a year and is a common assumption in economic analysis when including maintenance. Nonetheless, SL was expressed in some cases in m^3^ treated, to simplify the interpretation of the graphs. In other scenarios, the default SL of NF-270 was considered 10 years.

#### 2.2.3. Life-Cycle Inventory Data Collection

The LCI of the replacement, recycling impact and impact of membrane production were obtained from the literature [18,28,29]. The impact of the production of a new commercial NF-270 was adapted from an existent inventory [30]. The main modification was the substitution of a CFC solvent with hexane, as suggested in [31]. Euro 6 lorries were considered for transport processes and landfilling was modelled according to [28,29]. SECs included in the assessment varied depending on the scenario; they are discussed in the Results and Discussion section (Section 3). SEC was modelled following the procedure described in Section 2.3 and the Supplementary File. To treat the epistemological uncertainties in prospective LCAs of emergent energy-related products such as r-NF, scenario analysis using different background processes is the best methodological framework [33]. The European electricity mix (EU-27) was used as a baseline. However, the near future of energy is decarbonisation. Therefore, alternative sources of energy are being implemented in the water-energy nexus. Due to the relevance of energy in this assessment, a sensitivity assessment was performed to change the electricity source. As an alternative, natural gas combustion plants and two different renewable energies were also studied: solar photovoltaic and wind energy. Finally, a GD scenario was included. It represents the inclusion of a membrane system using a flow in which mechanical energy and inertia were not used [24]. 

#### 2.2.4. Life-Cycle Impact Assessment Category 

Climate change is one of the most important environmental crises that humanity faces. Energy consumption is highly related to this phenomenon due to the high remaining contribution of fossil fuels to its production. Therefore, GWP (CF indicator) was the most suitable Life Cycle Impact category to illustrate the life cycle perspective of the membranes as energy-related products. Characterisation factors from IPCC 2013 with a horizon time of 100 years were used [34].

### 2.3. Specific Energy Consumption (SEC) Modelling 

A single-stage water treatment process was modelled. 

The functionality of an r-NF system is defined by the amount and quality of water that can be produced from a certain flow (Q_in_). Therefore, the design of the systems was defined by its Q_in_ and WR. Then, for a given Q_in_, WR, *n* and type of element (r-NF or NF-270), Pr_in_ and SEC were estimated. *n* in this stage was constrained between 1 to 16, considering one or two PVs of one to eight elements per PV. In each case, modelling was performed assuming a unique PV.

SEC is defined by the unitary energy use per m^3^ treated (kwh·m^−3^). It is estimated through Equation (2), where Energy is the energy consumed by the pumping system (in kwh) and Q_pr_ is the product flow (in m^3^·h^−1^)

(2) $$\mathrm{SEC}=\frac{\mathrm{Energy}\;}{Q_{\mathrm{pr}}}$$

The energy use of pumping was estimated through Equation (3), where Q_in_ is the inlet flow (in m^3^·h^−1^) and Pr_in_ is the inlet pressure (in Pa = Bar·10.2^−1^). The efficiency factor (Ef) was assumed to be 0.75 and the density (ρ) equal to 1000 kg·m^3^; the gravity factor (g) is 9.8 m^2^·s^−1^.

(3) $$\mathrm{Energy}=\frac{Q_{in}{\cdot \;\rho \;\cdot \;g\;\cdot \;\mathrm{Pr}}_{in}}{\mathrm{Ef}\;\cdot 3600\cdot \;1000}$$

WR can be defined as the proportion of inlet water that is transformed into the product (Equation (4)). Mass and energy balances were maintained as described in Equation (S.1) (Supplementary File).

(4) $$\mathrm{WR}=\frac{Q_{pr}\;}{Q_{in}}$$

Pressure losses within a PV (Prl_total_) can be estimated through Equation (5), where Prl_in_ is the pressure losses at the feed flow (in bar) and Prl_conc_ is those at the concentrate flow, and n is the number of modules, which corresponds to length (in m) because their length is 1 m. Prl_in_ and Prl_con_ were estimated following the equations in the Supplementary File Equations (S.5)–(S.8)

(5) $${\mathrm{Prl}}_{\mathrm{total}}=\frac{{\mathrm{Prl}}_{\mathrm{in}}{+\mathrm{Prl}}_{\mathrm{conc}}}{2}\cdot n$$

The Net Pressure (NPD) needed for the obtention of the target flow was calculated according to Equation (6), where L is the permeability.

(6) $$\;\mathrm{NPD}=\frac{{\;Q}_{\mathrm{pr}}\cdot 1000}{L\cdot n\cdot a}$$

Finally, the Pr_in_ required was estimated using Equation (7), where the product pressure (Pr_pr_) was assumed to be 0.

(7) $${\mathrm{Pr}}_{\mathrm{in}}{=\mathrm{NPD}+\mathrm{Pr}}_{\mathrm{pr}}+\frac{{\;\mathrm{Prl}}_{\mathrm{total}}}{2}$$

The osmotic pressure (П) was not considered. Although it can be a relevant contributor to SEC, it was excluded to simplify the analysis. It was considered that for a given design (number of elements, flow, and WR) the polarization effect occurs in both membrane types; thus, it does not affect the comparison [27]. Another aspect would be the selectivity of different ions, which can alter the polarisation effect. However, this is outside the scope of our study. 

Modelling results with NF-270 (mass balance, energy balance and SEC) were compared with modelling in Wave^®^ software. SEC results varied by less than 1 % and mass and energy balances were correlated, with R^2^ > 0.995. Therefore, the model was considered validated.

### 2.4. Case Study Description

For the case study, an EoL-BWRO membrane recycled into NF (r-NF) was compared with a commercial, newly produced 8′ spiral wound NF-270 membrane. These EoL-BWRO membranes were recycled at a pilot scale with a NaClO dose of 6,000 ppm·h and, then, characterised as reported in García-Pacheco et al. [14]. Both membranes (NF-270 and r-NF) were tested and characterised when treating natural BW from a well. For this study, pure water L was estimated to be used later on in energy modelling. Thus, the tested BW permeabilities were corrected, with the П using the Van ’t Hoff equation (see Supplementary File Equation (S.9)). The estimated pure water L of NF-270 and r-NF were 10.5 L·m^−2^·h^−1^·bar^−1^ and 7.3 L·m^−2^·h^−1^·bar^−1^, respectively. The value of NF-270 coincided with that reported in Wave^®^ software. The modelling conditions were Q_in_= 4 m^3^·h^−1^, WR = 0.5 and n = 8. With Strategy A, both systems had n = 8. However, with strategy B, scenario NF-270 and n = 8 were compared with r-NF and n = {8…16}.

### 2.5. Technological Niche Exploration via Analytical–Numerical Method and LCA-Based Indicators

The novel hybrid analytical method developed herein combines the analytical resolution of a mathematical problem with numerical simulation. For this case, the minimum permeability ratio and minimum service life ratio were estimated analytically (Section 2.5.1). These estimations were performed for a wide range of design conditions (Section 2.5.2), and both strategies were analysed. 

#### 2.5.1. Life Cycle Assessment-Based Indicators

The use of LCA-based indicators is based on the identification of boundary conditions, where decision-making can experience a turn between one or multiple systems. The most relevant point to find, in our case study, was the equilibrium between the impact of r-NF systems (indexed with the subscript r) and NF-270 systems (indexed with the subscript c), as shown in Equation (8).

(8) $${\mathrm{Imp}}_{\mathrm{rep},r}{+\mathrm{Imp}}_{e,r}{=\mathrm{Imp}}_{\mathrm{rep},c}{+\mathrm{Imp}}_{e,c}$$

The first indicator developed was the service life ratio (SLR). This is defined in Equation (9). For the simulations, the service life of the commercial membranes (SL_c_) was assumed to be 10 years.

(9) $$\mathrm{SLR}=\frac{{\mathrm{SL}}_{r}}{{\mathrm{SL}}_{c}}$$

The estimation of SLR was developed using (Equations (S.10)–(S.14)—Supplementary File). The result is Equation (10).

(10) $$\mathrm{SLR}=\frac{1}{\left(\frac{{\mathrm{Imp}}_{\mathrm{rep},c}}{{\mathrm{SL}}_{c}}\cdot \left({\mathrm{SEC}}_{c}{-\mathrm{SEC}}_{r}{)\cdot \mathrm{Imp}}_{e}\cdot \frac{Q_{pr}\cdot 6000}{n}\;\right)\right)}\cdot \;\frac{{\mathrm{Imp}}_{\mathrm{rep},r}}{{\mathrm{SL}}_{c}}\;$$

The second indicator estimated was the permeability ratio (LR), which is defined in Equation (11).

(11) $$\mathrm{LR}=\frac{L_{r}}{L_{c}}$$

For estimation of the required permeability of r-NF (L_r_ in L·m^−2^·h^−1^·bar^−1^) to achieve the abovementioned equilibrium, firstly, SEC_r_ was estimated, to compensate for the low impact of the replacement (Equation (S.15)). Then, the pressure required for the obtention of SEC was estimated (Equation (S.16)). Finally, L_r_ was estimated with an equation system developed from the abovementioned equations (Equations (3)–(10), (12) and (S.17).

(12) $$L_{r}=\frac{Q_{\mathrm{pr}}\cdot 1000}{{\mathrm{NPD}}_{r}{\cdot n}_{r}\cdot a}$$

#### 2.5.2. Numerical Simulation: Framework Conditions

The abovementioned analytical method (Section 2.2.1) was executed under different ranges and combinations for both strategies:

1. Water flow (Q_in_ in m^3^·h^−1^): {1..16}
2. Number of elements per pressure vessel (n): {1..16}
3. Recovery rate (WR): [0.1, 0.95]
4. Energy sources: Mix, Natural gas, Solar and Wind

For analysis of strategy A, the number of modules did not vary (n_r_ = n_c_). However, for the case of strategy B, the number of modules varied (n_r_ > n_c_). For both strategies, n_c_ = 8 was assumed to be the central scenario. Similarly, WR = 0.5 was presented in the core results section of the article. 

Manufacturers recommend a maximum 15% water recovery rate per module [35]. Nonetheless, this was not considered a constraining factor. It is discussed as a rule, and there are examples of higher flow rate drops [36]. Another aspect recommended by manufacturers is a concentrated flow (Q_conc_) higher than 2 m^3^·h^−1^, as high as 3.5 m^3^·h^−1^ depending on the solute profile [37]. This limitation aims to guarantee a minimum flow velocity (v). However, we considered it interesting to see the environmental profile under a variety of flows. 

### 2.6. Software, Hardware, and Databases 

Life Cycle Impact Assessment was performed using Brightway v2.4 [38] and Ecoinvent v3.8 cut-off database [39] in a Jupyter notebook with Python v3.9 [40]. The category GWP 100 years from IPCC 2013 was used for carbon footprinting [41]. The script and Excel database were also in the Mendeley dataset. The modelling and data analysis were performed in R v4.1 with R-Studio [42]. *Tidyverse* was used for tidying and *ggplot2* for the graphs [43,44]. All applications were run in an HP EliteBook with an 11^th^ Gen Intel^®^ Core ™ i5-1135G7 of 2.40 GHz and 16.0 GB of RAM with Windows 10 (64 bits). 

## 3. Results and Discussion

The results section is divided into two subsections: (i) presentation of the strategy results using r-NF and NF-270 membranes, and (ii) results of the novel hybrid analytical-numerical method developed to identify the thresholds.

### 3.1. Case Study: Concept Illustration of Strategy A and Strategy B

**Figure 2:**
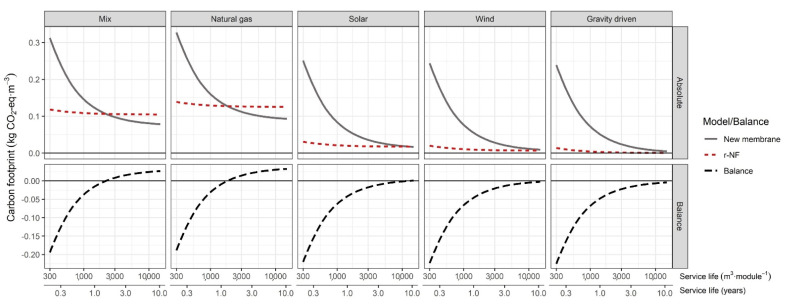
**C**arbon footprint and balance depending on membrane lifespan under strategy A. Conditions: Q_in_ = 4 m^3^·h^−1^, WR = 50% and n = 8.

Strategy A compensates for lower permeability with increments in the inlet pressure. Figure 2 illustrates the CF results for r-NF and NF-270 when using the following parameters: Q_in_ = 4 m^3^·h^−1^, WR = 0.5 and n = 8. The lines illustrate the CF of the treatment process (production of 1 m^3^ nano-filtered water) depending on the SL of the membranes (in years and m^3^) and for different energy sources.

Pressure, and thus SEC, increased in the case of r-NF (as summarised in Table 1). These were closely related to the LR between the r-NF and NF-270 membranes (over 0.7), as expected. The SEC increment of 42% had a great contribution to the environmental impact. The r-NF impact was almost a horizontal line when using the central scenario (EU-27 energy mix as background processes). The main contribution to overall CF was the energy required for the filtering process (99% with SL = 10 years). Membrane replacement had a low impact, almost negligible. This was because the impact of the replacement of the eight membranes was distributed. By contrast, the NF-270 system had a marked reciprocal function shape. That represents an elevated impact of membrane replacement that was distributed across a greater volume of product water when the lifespan was lengthened. For instance, the replacement of eight r-NF modules was 33.28 kg CO_2_-eq. (r-NF: 4.16 kg CO_2_-eq. module^−1^). However, the impact of the replacement of eight commercial NF-270 elements was 574.4 kg CO_2_-eq. (71.8 kg CO_2_-eq.·module^−1^). The difference was 541.12 kg CO_2_-eq., which means a reduction of 94% when membrane replacement used recycled membranes. Nonetheless, the SEC variance generated only a small difference of 0.031 kg CO_2_-eq·m^−3^ in favour of commercial membranes. Therefore, the savings on replacement when using recycled membranes were compensated for by a higher SEC, which meant that neutrality (compared with commercial membranes) was achieved when producing 2,200 m^3^·module^−1^ or 17,600 m^3^ for the whole eight-element system. Neutrality occurred at the crossing point between both CF lines (r-NF and NF-270). For a better extrapolation of the results, the SL was described in m^3^ and hours, considering 6,000 working hours·year^−1^. Neutrality determined the SL under which r-NF modules (with 7.3 L·m^2^·h^−1^·bar^−1^) had a potential technological niche with a greater environmental outcome (at least for the CF indicator of the GWP category). In this context, r-NF would be a preferred option over commercial NF membranes in processes with a membrane SL below 1.46 years. Therefore, the longer the SL crossing point is, the wider the applicability of the r-NF membranes will be.

In scenarios with low carbon emissions energy sources (solar and wind), the overall impact of both systems (r-NF and NF-270) reduced drastically. It is known that energy source choice has a great influence on the overall impact of several membrane processes [45]. The crossing point of neutrality (Imp_total,c_ = Imp_total,r_) shifted significantly towards longer lifespans: 36,000 m^3^·module^−1^ or 24 years with wind energy and 13,500 m^3^·module^−1^ or 9 years with photovoltaic solar energy. This reinforces the position that within the technological niche, substitution with r-NF membranes could be favoured the process decarbonisation. Compared with newly produced membranes, they have important carbon savings below the SLs indicated. The coupling of membrane technology with renewable energy sources has been studied in depth since the beginning of the technology [46]. However, technical complexity exists in the creation of systems isolated from the grid. By contrast, the use of natural gas sources had a higher CF than the EU-27 mix, so the crossing lifespan was the opposite: 1.22 years or 1830 m^3^. 

**Figure 3:**
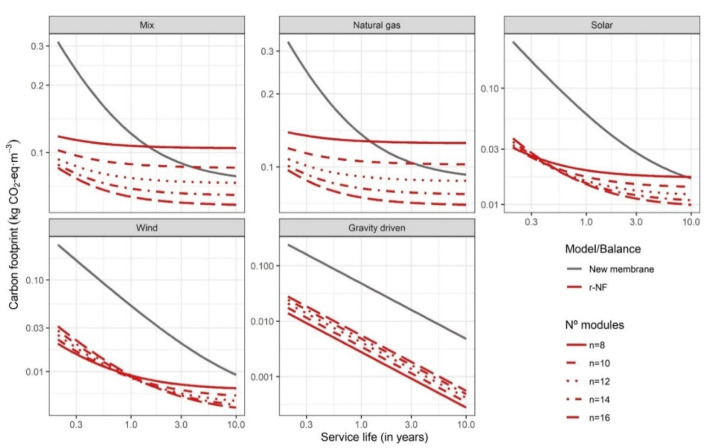
Carbon footprint depending on PV design and membrane lifespan with strategy B. Conditions: Q_in_ = 4 m^3^·h^−1^ and WR = 50%.

Strategy B compensates for the lower permeability with increments in the number of modules included in the stage. Figure 3 illustrates the CF results of this second strategy and Table 1 summarises the Pr_in_ and SEC modelling, as well as the SL when the impact of the NF-270 system is equal to that of the r-NF system. It can be observed that the increment in modules could mitigate low permeability and reduce SEC drastically by increasing the active area. The SEC reduction was almost proportional to the increment in modules. In the case study, SEC equal to that of the NF-270 system (n = 8) was achieved with 12 modules. The modelling confirms that the relationship of 12/8 (1.5) corresponds similarly to the inverse relationship in the r-NF permeability ratio (0.69), as expected. That is, PR^−1^ = 0.69^−1^ = 1.45. The differences were below 5%. Increments exceeding 12 recycled elements reduced the SEC of the recycled membranes below the SEC of NF-270, which, together with the low replacement impact, entails a lower impact than NF-270. However, the advantage of the use of a higher number of elements should be analysed economically. Previous works pointed out a potentially lower cost for r-NF modules (product cost of 80–120 EUR·module^−1^) [18,47]. The identification of limits besides permeability and the relationship with a reduction in the use of commercial modules with higher prices should be further assessed. Nevertheless, this analysis should include CAPEX extra costs, such as longer PVs or a greater number of PVs, and other infrastructure elements that can have a non-negligible cost.

Secondly, a minimum increment (below PR^−1^) to partially compensate for SEC could be enough to have a lower impact in most circumstances (Table 1). Particularly, it is important to note the synergies between compensation for lower permeability and the use of low-carbon energy sources. However, with very short lifespans using renewable energies (solar: <0.3 years, wind: <1 year), the order of the number of modules is inverse, similarly to the case of the GD system throughout all SLs. This is because the use of more modules in the design, even with a lower individual impact as in the case of r-NF systems, can result in a higher total impact than that avoided by SEC reduction. Nonetheless, GD scenarios should be treated with nuance. The higher impact with additional modules complicates the placement of systems due to the need for higher unused pressured flows. By contrast, the increment in the number of modules could favour placements reducing the pressure requirements of these systems. This hypothesis is also confirmed by results using different Q_in_, n, and WR, represented in Figure S2 in the Supplementary File.

### 3.2. Hybrid Analytical–Numerical Modelling

The application of a hybrid analytical-numerical method, in combination with the use of LCA-based indicators such as SLR and PR, allows for exploration of the suitability and potential of each strategy. To ease interpretation of the layout, the core of the manuscript compares analyses with WR = 0.5. 

#### 3.2.1. Application of the Hybrid Analytical–Numerical Method in Strategy A

**Figure 4:**
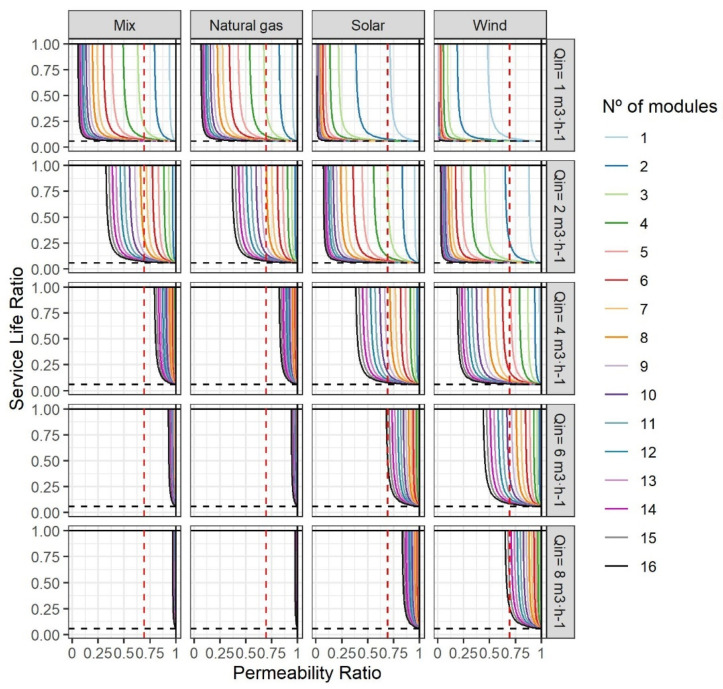
Results of the hybrid analytical–numeric modelling approach for r-NF with Strategy A. Reference scenario for NF-270: SL = 10 years and WR = 0.5. The dashed red line illustrates LR from the case study (0.69) and the dashed black lines the ratio of replacement impact (0.0579).

Figure 4 summarises the functions obtained from the application of the analytical method to a wide range of conditions (numerical modelling). The lines illustrate the relationships between SLR and LR for different design conditions (n and Q_in_ with WR equal to 0.5) and energy sources under strategy A (n_c_ = n_r_). Furthermore, the approach was carried out using the four energy sources mentioned above. The results for GD were excluded from this part. The limits found were low, and LR limits were associated with limits in the replacement and the relationship between replacement impacts.

Firstly, the shapes of the curves in the graphs highlight the relevance of LR (with PR) in the definition of feasible designs. The verticality of the lines (depending on n) is the main indicator and means that there was a minimum LR below which r-NF membranes had a greater impact than commercial NF membranes. Depending on Q_in_, LR wideness reduced drastically. That reflects the relevance of SEC in the overall impact of the r-NF membrane life-cycle due to permeability, as evidenced in the literature [21,22,23,24]. By contrast, SLR was limited with a horizontal asymptote (at the value of 0.0579). That is the relationship between the replacement impacts mentioned above. This reinforces the idea that, for NF processes with low membrane lifespans, the use of r-NF membranes with lifespans above six months (in comparison with 10 years for commercial counterparts) could be an option if LR is within the burdens illustrated in Figure 4. Some such processes would be harsh waters with significant potential to damage the membrane surface and selectivity properties (i.e., industrial wastewater treatments with solvents) or with high fouling rates (i.e., landfill leachate treatments, manure treatments or zero liquid discharge) [48,49,50,51,52,53,54]. 

Strategy A could be defined as an analysis of the design and operational conditions, as well as the minimum requirements, of r-NF that have to replace pre-existing NF-270 modules. Firstly, it should be remarked that Q_in_ below 4 m^3^·h^−1^ would be difficult to find because that is the minimum flow requirement recommended by manufacturers. Therefore, although under low flows r-NF are more competitive in environmental terms, the main scenarios to focus on should be high flows with the current energy mix. In these scenarios, a reduction in the LR range could be observed as the inlet flow increased. The need for high inlet flows is normally associated with the prevention of fouling phenomena [29]. The higher the inlet flow, the higher the energy used for pumping. Therefore, low permeabilities demand a more elevated inlet pressure that synergistically increases SEC, as described in Equation (2). Another aspect not covered in the present article is the existence of energy recovery devices (ERD). These systems are common in the NF process. For instance, with a Q_in_ over 8 m^3^·h^−1^ and eight elements per PV, SEC reduction could reach 46% by combining an ERD with a second stage [55]. As observed in the present results, the SEC reduction in both systems would favour the use of r-NF membranes. However, modelling under different conditions would also allow for quantification of the impact on niche expansion.

Another evidenced trend was the relationship between systems with a higher n that mitigates the inlet pressure-demand, and thus SEC, in both systems. For instance, the abovementioned r-NF membrane with an LR equal to 0.69 would be unrecyclable compared with NF-270 membranes with an SL_c_ of 10 years. Of course, as discussed in Section 3.1 and the Supplementary File, other referent values would vary the results of the modelling. In any case, PR values above 0.75 would exclude as recyclable an important number of r-NF modules evidenced in the literature [18]. The estimated LR values from the literature are summarised in Figure S1 (Supplementary File). The extrapolation of the hybrid method should be relevant to other pressure-driven recycled membrane technologies, such as r-UF and recycled membranes for membrane bioreactors [10,56]. These technologies have lower SEC than NF and, therefore, permeability could have a lower contribution to the overall impact and their application could be wider [57]. Moreover, in the case of spiral-wound r-UF membranes, LR was proven to be close to 1, which would also signify more possibilities to find modules with high LR [18].

#### 3.2.2. Application of the Hybrid Analytical–Numerical Method in Strategy B

**Figure 5:**
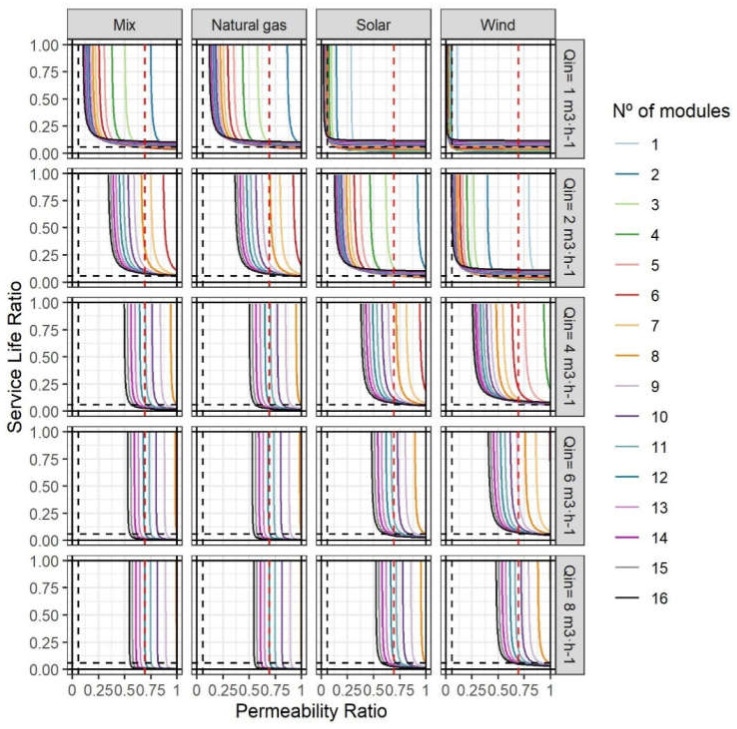
Results of the hybrid analytical–numeric method for r-NF with Strategy B (n_c_ ≠ n_r_). Reference scenario for NF-270: SL = 10 years, n_c_ = 8 and WR = 0.5. The number of modules refers to n_r_. The dashed red line illustrates LR in the case study (0.69) and the dashed black lines the ratio of the replacement impact (0.0579).One difference compared with strategy A is the limitation in the minimum SLR, which varied depending on the contribution of the replacement. With low flows and with non-fossil-based energy sources, the minimum SLR depended on *n*. However, with a highly energy-dependent scenario, that SLR could be compensated for up to almost 0 by a lower SEC than NF-270 systems.

Figure 5 summarises the results of the application of the hybrid analytical-numerical method in strategy B. It is relevant to highlight that these results compare different operational conditions and the use of different numbers of modules to the reference scenario of NF-270 with n = 8. A common point between Figure 4 and Figure 5 is n = 8. This maintains the principle of strategy A by using an equal number of modules (n_c_ = n_r_). The most relevant aspect is to see whether increments in the number of elements entail a wider range of LR values. Lower permeabilities of r-NF modules could be easily compensated for by this design change. Of course, this strategy could be difficult to apply in the case of existing systems due to the need to change the PVs. However, the design of new systems allows increments in the number of elements. When doubling the n, the estimated LR was half. That indicates quasi-perfect maintenance of the theoretical ratios, even with the modelling of pressure losses. Outside scenarios with low feed flow (Q_in_ = 1–2 m^3^·h^−1^), indicate a lower environmental impact from r-NF membranes than commercial counterparts. These flows are not recommended by the manufacturers of commercial NF membranes because of the fouling risk. However, previous papers indicated the possibility that surface negativity charges could be developed from the recycling process, which could have antifouling properties [10]. Therefore, it would be interesting to validate r-NF with low flows with different effluents. 

As mentioned in Section 3, synergies between the use of r-NF and renewable energies favour carbon-neutral membrane processes. Furthermore, the coupling of membrane technology powered by renewable energy was pointed out as a solution for secure water access in rural areas [45]. Nonetheless, other strategies should be analysed, such as energy use with hourly discrimination. Previous articles, applying a consequential approach, identified important differences in the definitions of marginal suppliers that could satisfy the increased energy demand due to new r-NF systems [44]. For this purpose, the consequential perspective could be more accurate, identifying the hourly marginal suppliers [44].

Finally, the present article assessed a single LCA indicator (GWP or CF) to ease the interpretation of the results and illustrate the conceptual framework. Further studies should complete the vision for r-NF membranes as well as other abovementioned membrane products by using other impact categories. Other categories could display different behaviours and identify more limitations or advantages of recycled membranes. For instance, according to Senán-Salinas et al., ozone depletion or marine eutrophication categories could be more limiting than CF in the replacement of r-NF membranes [18,58]. This could result in a more restrictive SLR value. Moreover, renewable energies, although identified with a low CF, could have relevant impacts on other indicators, further constraining the use of r-NF membranes with higher LR values. Some of these categories could include non-fossil resource depletion due to the use of several scarce minerals in their production stage. In addition, energy use from the EU-mix could have a high influence on other ILCD categories, as ionisation radiation categories related to nuclear power or human toxicity categories were also identified as limiting the recycling supply chain in previous works [18,26,42,58]. Nonetheless, the application of the hybrid analytical–numerical model to additional impact categories could be difficult to understand. It would also need a further step for summarising and skimming the data results. That implies the use of different tools to assess multicriteria in the decision-making as well as data interpretation and plotting. Moreover, economic feasibility could be another complementary indicator for establishing the market niche and the recyclability of recycled membranes from a life-cycle perspective. 

## 4. Conclusions

For the first time, the present article analyses the SEC and CF of r-NF membranes as a potential water treatment case. Moreover, as a novelty, a hybrid analytical–numerical mathematical method with LCA-based indicators was developed to identify optimal environmental design conditions and recyclability thresholds for r-NF membranes compared with their commercial counterparts (NF-270). The results clearly show the relevance of the permeability of r-NF to the definition of the operational conditions and design, particularly in substitution with r-NF using the same number of elements (Strategy A). Nonetheless, new treatment designs (Strategy B) with r-NF membranes could compensate for the low permeability with a higher number of elements in line. The coupling of r-NF with renewable and non-fossil-based energies reduces the overall impact of NF processes in a greater way than the coupling of commercial NF modules with these low-carbon energy sources. Moreover, an assessment with GD systems also evidenced the possibilities of almost carbon-neutral processes for r-NF. 

The use of LCA-based indicators for assessment in decision-making as well as to seek the potential of a recycling system seems to be more flexible and less orthodox use of the LCA methodology. However, it provides important results and insights regarding the potential interactions of technology performance at low TRLs or with a prospective vision. The provision of realistic science-based indicators for assessing the CE transition is a critical question for guaranteeing environmental protection and avoiding undesired hidden impacts, although more LCA categories should be estimated.

## Figures and Tables

**Table 1:** Process Pr_in_, SEC and crossing service life for equivalent carbon footprint by energy source. Conditions: Q_in_ = 4 m^3^·h^−1^ and WR = 50%. Infinite values were marked as Inf.

System	N° of Elements	Pr_in_ (in bar)	SEC (in kwh·m^−3^)	Crossing Service Life (SL, in Years)				
				EU-27 Mix	Natural Gas	Solar	Wind	GD
NF-270	8	0.678	0.188	-	-	-	-	-
r-NF	8	0.962	0.267	1.46	1.22	8.98	24.3	-Inf.
	9	0.863	0.240	2.22	1.85	13.6	36.8	-Inf.
	10	0.785	0.218	3.79	3.16	23.3	62.9	-Inf.
	11	0.722	0.200	9.02	7.53	55.5	150	-Inf.
	12	0.670	0.186	<0	<0	<0	<0	-Inf.
	14	0.591	0.164	<0	<0	<0	<0	-Inf.
	16	0.533	0.148	<0	<0	<0	<0	-Inf.

## Data Availability

The data are available at the Mendeley dataset: https://data.mendeley.com/datasets/ryfb8h9rzj/draft?a=f16c525e-db48-49ba-9e2d-c573cbe51f88 (accessed on 29 July 2022).

## Supplementary Materials

The following supporting information can be downloaded at: https://www.mdpi.com/article/10.3390/membranes12090854/s1, Figure S1: Service Life in which the r-NF and NF-270 have the same impact title; Figure S2: Permeability ratios for r-NF modules; and Equations (S.1)–(S.17). Reference [18] is cited in the Supplementary Materials.

## Nomenclature

Symbol/Abbr.	Description
µ	Viscosity (in m^2^·pa·s^−1^)
a	Area (in m^2^)
BW	Brackish water
CE	Circular Economy
CF	Carbon footprint (in kg CO_2_-eq)
CFC	Chlorofluorocarbon
Cl^-^	Chloride
dH	Hydraulic diameter (in mm)
Ef	Efficiency factor
EoL	End-of-life
ERD	Energy Recovery Device
EU	European Union
g	Gravity force
GD	Gravity-driven
h	Hours
i	Van ’t Hoff index
ILCD	International Life Cycle Data system
Imp	Environmental impact (in kg CO_2_-eq)
Inf.	Infinite
IPCC	Intergovernmental Panel on Climate Change
ISO	International Standard Organization
L	Permeability (in L·m^−2^·h^−1^·bar^−1^)
L (unit)	Litre
LCA	Life Cycle Assessment
LR	Permeability ratio
m	Metre
M	Molarity
n	Number of modules
Na^+^	Sodium ion
NaClO	Sodium hypochlorite
NF	Nanofiltration
NPD	Net Pressure Driven (in bar)
ppm	Parts per million
Pr	Pressure (in bar)
Prl	Pressure loss (in bar)
PV	Pressure vessel
Q	Flow (in m^3^·h^−1^)
R	Ideal gas constant
Re	Reynolds number
r-NF	Recycled nanofiltration
RO	Reverse osmosis
SEC	Specific energy consumption (in kwh·m^−3^)
SL	Service Life (in years)
SLR	Service life ratio
SW	Seawater
T	Temperature (in K)
TRL	Technology Readiness Level
UF	Ultrafiltration
v	Velocity (in m·s^−1^)
ν	Kinematic viscosity
vs.	Versus
WR	Water recovery rate
П	Osmotic pressure (in bar)
**Subscript**	
Subscript	Description
c	Relative to new commercial produced membranes
conc	Relative to concentrated water flow
e	Relative to energy
in	Relative to inlet water flow
pr	Relative to product water flow
r	Relative to recycled membranes
rep	Relative to replacement activity
total	Relative to the total of the system
