# Supplementary material for: Recyclability Definition of Recycled Nanofiltration Membranes through a Life Cycle Perspective and Carbon Footprint Indicator
Source: Membranes (Basel). 2022 Aug 31;12(9):854. doi: 10.3390/membranes12090854 (PMC9505957; doi:10.3390/membranes12090854)
Supplement: Supplementary file 1 [file membranes-12-00854-s001.zip › membranes-1867721-supplementary.pdf]

Supplementary Material

Recyclability Definition of Recycled Nanofiltration Membranes through a Life Cycle Perspective and Carbon Footprint Indicator

Jorge Senán-Salinas [1,2,3,*], Junkal Landaburu-Aguirre [1], Raquel García-Pacheco [4] and Eloy García-Calvo [1,2]

1 IMDEA Water Institute, Avenida Punto Com, 2, Alcalá de Henares, 28805 Madrid, Spain
2 Chemical Engineering Department, University of Alcalá (UAH), Ctra. Madrid-Barcelona Km 33.600, 28871 Alcalá de Henares, Spain
3 BETA Technological Center, University of Vic-Central University of Catalonia, Ctra. De Roda, 70, 08500 Vic, Spain
4 Laboratory of Chemical and Environmental Engineering (LEQUIA), Institute of the Environment, University of Girona, 17003 Girona, Spain
* Correspondence: jorge.senan@uvic.cat; Tel.: +34 93 881 61 68.

A.-Service Life Ratio & Permeability Ratio development

$$\mathrm{Imp}_{\mathrm{rep,c}}=\frac{\mathrm{n_c}\cdot(\mathrm{Imp_c}+\mathrm{Imp_{land,\,c}})}{\mathrm{SL_c}\cdot\mathrm{Q_{pr}}\cdot 6000} \quad \text{(S1)}$$

$$\mathrm{Imp}_{\mathrm{rep,r}}=\frac{\mathrm{n_r}\cdot\mathrm{Imp_r}}{\mathrm{SL_r}\cdot\mathrm{Q_{pr}}\cdot 6000} \quad \text{(S2)}$$

B.-Energy Modelling

$$\mathrm{Q_{in}}=\mathrm{Q_{pr}}+\mathrm{Q_{conc}} \quad \text{(S3)}$$

$$\mathrm{Pr_{in}}=\mathrm{Pr_{conc}}+\mathrm{Prl_{total}} \quad \text{(S4)}$$

For a given position within the PV, the linear velocity (v_{in} in $m\cdot s^{-1}$) can be estimated as in Equation S5 where dh is the hydraulic diameter (dh= 9 mm)

$$\mathrm{v_{in}}=\frac{\mathrm{Q_{in}}\cdot\mathrm{dh}}{3600\cdot\mathrm{section}} \quad \text{(S5)}$$

Where section in a spiral wound module can be defined as in Equation S6 where the area (a) is equal to 37 m^2

$$\mathrm{section}=\mathrm{dh}\cdot\mathrm{a} \quad \text{(S6)}$$

Re can be estimated as in Equation S7 where the ν is the kinematic viscosity (10^{-6} $Pa\cdot s^{-1}$).

$$\mathrm{Re}_i=\frac{\mathrm{v_i}\cdot\mathrm{dh}}{\nu} \quad \text{(S7)}$$

The pressure losses (Prl) were estimated as in Equation S8.

$$\mathrm{Prl_i}=6.23\cdot\mathrm{Re_i}^{-0.3} \quad \text{(S8)}$$

C.-Osmotic pressure

Equation S9 describes the Van´t Hoff equation where i is the Van´t Hoff factor, M the molar concentration (in mol·L^{-1}), R the ideal gas constant (0.08206L·atm·mol^{-1}·k^{-1}), and T the temperature (in K).

$$\Pi = i \cdot M \cdot R \cdot T \qquad \text{(S9)}$$

D.-Service Life Ratio & Permeability Ratio development

Equations S10, S11, S12, S13 & S14 develop the development of SLR indicator

$$SEC_r \cdot Imp_e + \frac{n_r \cdot Imp_{rem,r}}{SL_r \cdot Qp \cdot 6000} = SEC_c \cdot Imp_e + \frac{n_c \cdot Imp_{rem,c}}{SL_c \cdot Qp \cdot 6000} \qquad \text{(S10)}$$

$$(SEC_r - SEC_c) \cdot Imp_e = \frac{n_c \cdot Imp_{rep,c}}{SL_c \cdot Qp \cdot 6000} - \frac{n_r \cdot Imp_{re,\,r}}{SL_r \cdot Qp \cdot 6000} \qquad \text{(S11)}$$

$$SL_r = SL_c \cdot SLR \qquad \text{(S12)}$$

$$(SEC_r - SEC_c) = \frac{\left(\left(\frac{Imp_{rem,c}}{SL_c} - \frac{Imp_{rem,r}}{SL_c \cdot SLR}\right) \cdot \left(\frac{n}{Qp \cdot 6000}\right)\right)}{Imp_e} \qquad \text{(S13)}$$

$$SLR = \left(\frac{-1}{(SEC_r - SEC_c) \cdot Imp_e \cdot \frac{Qp \cdot 6000}{n} - \frac{Imp_{rem,c}}{SL_c}}\right) \cdot \frac{Imp_{rem,r}}{SL_c} \qquad \text{(S14)}$$

For the estimation of the L_r, firstly, the SEC_r was estimated to compensate for the low impact of the replacement (Equation S15)

$$SEC_r = SEC_c + \frac{n_c \cdot \frac{Imp_{rep,c}}{SL_c} - n_r \cdot \frac{Imp_{re,pr}}{SL_r}}{Q_{pr,r} \cdot 6000 \cdot Imp_e} \qquad \text{(S15)}$$

Then the pressure required for the obtention of the SEC was estimated (Equation S16).

$$Pr_{in,\,r} = \frac{SEC_r \cdot Q_{pr,r} \cdot Ef \cdot 3600 \cdot 1000}{Q_{in,r} \cdot \varrho \cdot g \cdot 10.2} \qquad \text{(S16)}$$

Finally, the required Net Pressure Driven by the recycled membrane ($NPD_{r)}$ was estimated Equation S17

$$NPD_r = \frac{Pr_{in,r} \cdot 2 - Prl_{total,r}}{2} - Pr_{pr} \qquad \text{(S17)}$$

E. Service life of equilibrium

The results of the numeric simulation to estimate the SL in which the impact of both membranes is the same are illustrated in Figure 3. With the current electricity mix and the natural gas, the substitution is quite constrained to low WR and a high number of elements in the design. It has to be remarked that in the Scenarios with Qin below 4 $m^3 \cdot h^{-1}$, the possibilities for r-NF modules increase importantly. Nonetheless, these low flows are not recommendable due to the low velocity and the increase of fouling phenomena. However, r-NF has not been tested in a wide range of flows. Being low flow processes a potential testing proof as well as fouling behaviour in different conditions.

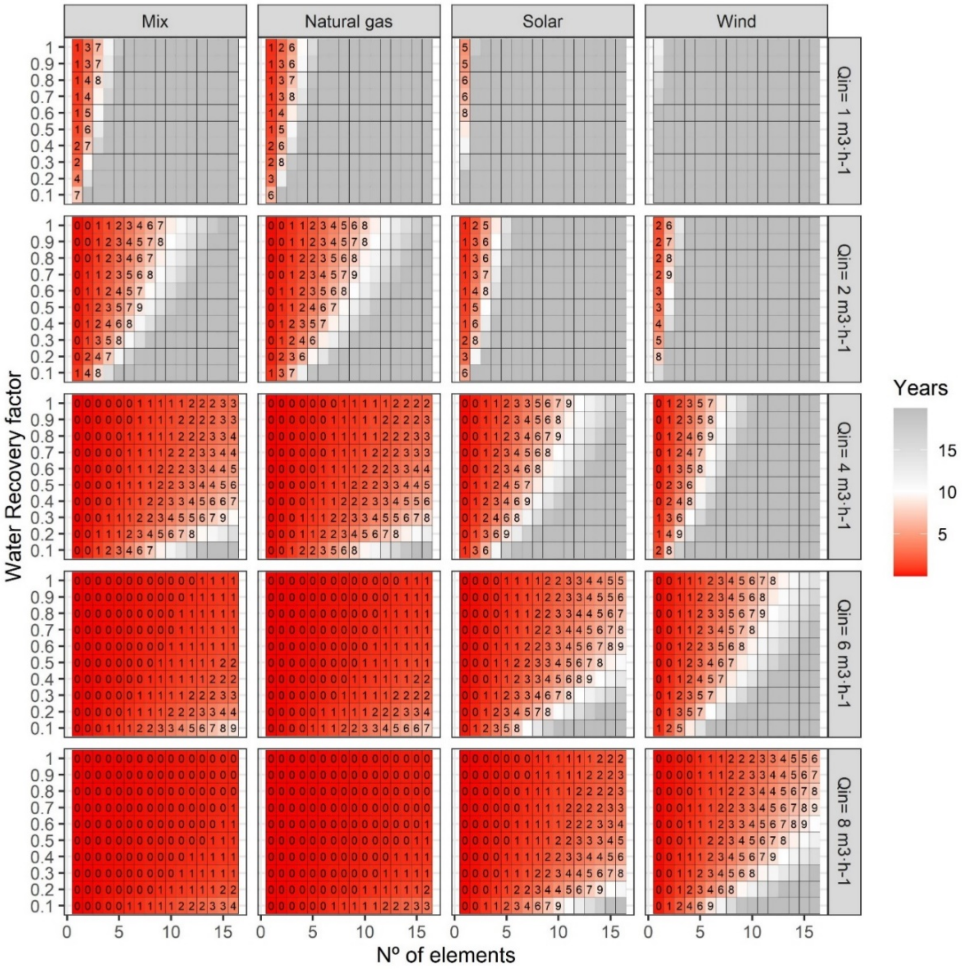

Figure S1. Service Life in which the r-NF and NF-270 have the same impact. An SL >=10 years has been considered as the threshold reference to consider the conditions favourable e to the r-NF.

F. Permeability ratios

Figure S2 illustrated the PRs obtained in [1].

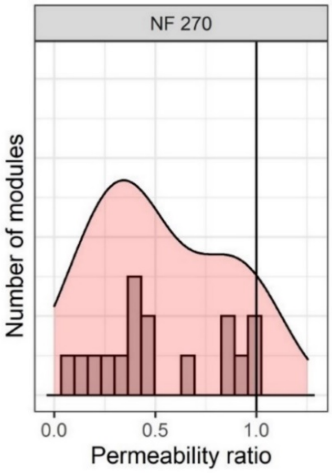

Figure S2. Permeability ratios for r-NF modules. Data from [1].

References

1. Senán-Salinas, J.; García-Pacheco, R.; Landaburu-Aguirre, J.; García-Calvo, E. Recycling of End-of-Life Reverse Osmosis Membranes: Comparative LCA and Cost-Effectiveness Analysis at Pilot Scale. *Resour. Conserv. Recycl.* **2019**, *150*, 104423, doi:10.1016/j.resconrec.2019.104423.
